# Scale morphometry, geometry and ultrastructure of three *Nemipterus* species from the Egyptian part of the Red Sea

**DOI:** 10.1038/s41598-025-30040-2

**Published:** 2025-12-15

**Authors:** Imam A. A. Mekkawy, Usama M. Mahmoud, Samia M. El-Mahdy, Ola I. Muhammad

**Affiliations:** 1 Zoology Department, Faculty of Science, Assiut University, Assiut, 71516 Egypt; 2 Fisheries Division, National Institute of Oceanography and Fisheries, NIOF, Hurghada, Red Sea Egypt

**Keywords:** Nemipterus, Scale characteristics, Scanning electron microscope, Morphometrics, Geometrics, Fisheries, Red sea, Ecology, Ecology, Evolution, Zoology

## Abstract

The present investigation aimed to document and analyze the diversity of scale characteristics in three *Nemipterus* species; *N. zysron* (12.2–18.5 cm SL), *N. randalli* (13–18 cm SL), and *N. japonicus* (8.5–17.2 cm SL) collected from the Egyptian Red Sea near Hurghada. Assessing interspecific differences in scale morphology, geometry, and morphometry provides valuable insights for taxonomy and stock identification. The results revealed pronounced interspecific variations in scale geometry, morphometrics, and radii meristics, particularly with respect to overall shape and size. Detailed structural features and surface ornamentation were examined using light microscopy and scanning electron microscopy. Notable differences were observed in surface morphology, interradial and intercircular grooves, interradial tongues and circuli, denticles, inner and outer lateral circuli and caudal field segmentation and granulation pattern. The observed variation in scale form among the three species underscores the potential utility of scale morphology in stock discrimination. Collectively, these findings contribute to improved species differentiation and offer a valuable tool for fisheries management and taxonomic assessment.

## Introduction

Fish scales, composed of ossified platelets, function as rigid external structures exhibiting complex morphological and structural characteristics. These features include scale type, shape, size, and specific surface ornamentations such as circuli, radii, lepidonts, and granules^1^. Because scales grow proportionally with the fish, new circuli are added at the periphery and remain preserved throughout the lifespan of the scale^2,3^. Scale shape is generally species-specific and has therefore been widely applied in elucidating fish systematics, determining stock structure, and assessing evolutionary relationships^4,5^. Considerable variability in scale morphology has been documented among different fish species, reflecting both intraspecific morphological plasticity and regional variation within individual specimens^3,6^. Such variation in scale shape may also contribute to reducing frictional drag during locomotion^1^.

In addition to their protective role against external injuries, certain diseases and predation, scales can influence swimming performance. Surface features such as ctenii, circuli, radii, and scale curvature affect the flexibility and rigidity of the scales, thereby influencing the magnitude of force required for body bending during locomotion and ultimately shaping overall swimming efficiency^2,5,7^.

Scale extraction is a non-lethal procedure that enables easy acquisition, preservation, and preparation for image-based analyses^1^. Owing to their distinctive morphological and structural characteristics, scales provide valuable information across a wide range of scientific applications, including fisheries assessment, age and growth determination, taxonomy, evolutionary and phylogenetic studies, archaeology and environmental monitoring^1–3,5,8–14^.

Numerous studies have applied a variety of methods to examine the morphometrics, geometric properties, morphological characteristics, and surface ornamentations of fish scales in both marine and freshwater species^1,3,4,6–8,11,15–20^.

The family Nemipteridae (threadfin breams) comprises approximately 77 species across five genera^21^ and is characterized by small to moderate body size and vibrant coloration^22^. Members of this family are primarily distributed throughout the tropical and subtropical Indo-West Pacific but are absent from the eastern Pacific and Atlantic Oceans^23^. The largest genus, *Nemipterus*^23^, includes many species of economic importance as a food source^24^. Extensive research on *Nemipterus* has addressed its biology^24–29^, population dynamics and fisheries^30–33^ as well as its biochemistry^34^ species identification and genetic diversity^23,35–37^.

Since the opening of the Suez Canal, the Mediterranean Sea has become connected to the Red Sea, facilitating a substantial influx of tropical fauna into the predominantly eastern Mediterranean basin. Numerous Indo-Pacific species (Lessepsian migrants) have consequently established populations and expanded their ranges within the region^38^. *Nemipterus japonicus* and *N. randalli* have been documented in the Mediterranean Sea^39,40^.

Threadfin breams constitute an important component of coastal demersal fish communities and regional fisheries^41,42^. In recent years, they have emerged as a significant fishery resource in Egyptian waters, particularly in the Suez Gulf trawl fishery, where their catch now accounts for approximately 7% of the total trawl yield^43^.

Despite the economic importance of *Nemipterus* species in Egypt, their scale characteristics have been relatively understudied. The present study aimed to investigate scale traits that can differentiate three nemipterid species; *Nemipterus zysron*, *N. randalli*, and *N. japonicus* from the Red Sea near Hurghada. Scales were examined using light and scanning electron microscopy, and univariate and multivariate analyses were applied to assess morphometric and geometric variations in size and shape.

## Materials and methods

### Specimen collection

A total of 601 scales were collected from three *Nemipterus* species: *N. zysron* (12.2–18.5 cm standard length), *N. randalli* (13–18 cm standard length), and *N. japonicus* (8.5–17.2 cm standard length). Specimens were obtained from fish markets in Hurghada (27° 15′ N, 33° 48′ E; Red Sea, Egypt) between September 2021 and August 2022.

### Preparation and measuring of scales

**Fig. 1:**
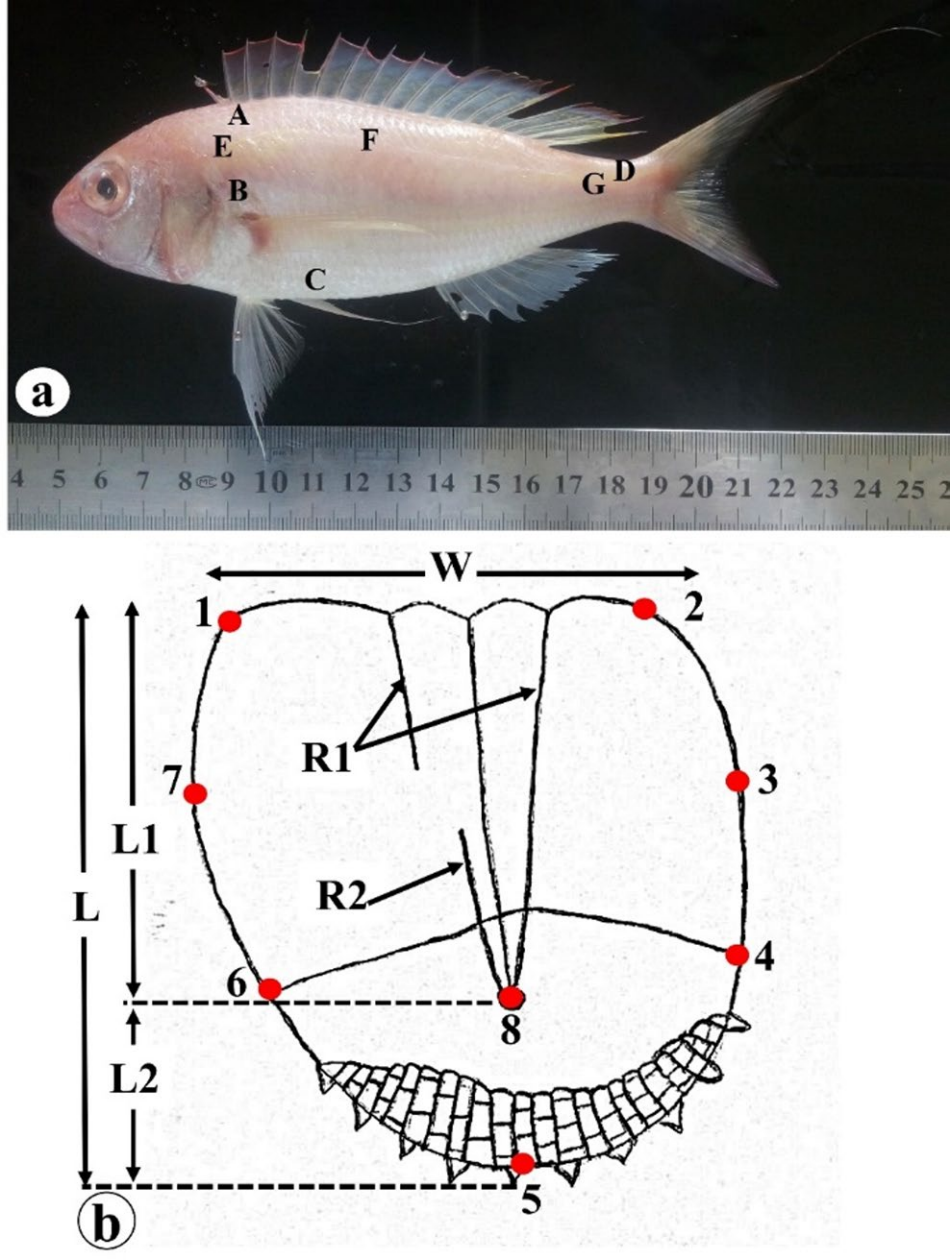
(**a**) Photograph of *N. randalli*, a representative of the studied species, illustrating the body regions from which scales were collected (A: BDFS, B: POS, C: BLLS, D: CPS, E: ALLS, F: MLLS, G: PLLS). (**b**) Schematic representation of a scale illustrating the primary radii (R1), secondary radii (R2), morphometric measurements (W: scale width, L: scale length, L1: rostral field length and L2: caudal field length) and landmark positions.

Scales were carefully extracted from the left side of the body, targeting seven specific anatomical regions as shown in Fig. 1a: (A) beneath the anterior portion of the dorsal fin (BDFS), (B) post-operculum (POS), (C) beneath the lateral line, between the pectoral and pelvic fins (BLLS), (D) caudal peduncle (CPS), (E) anterior lateral line (ALLS), (F) middle lateral line (MLLS), and (G) posterior lateral line (PLLS). Extracted scales were thoroughly cleaned in a 10% ammonia solution for 24–36 h to remove adhering tissue without compromising surface microstructure. Following cleaning, scales were dried on filter paper and mounted between two glass slides for storage.

Scales obtained from the first four regions were used to count primary (R1) and secondary (R2) radii and to obtain morphometric measurements, including scale width (W), caudal field length (L2), rostral field length (L1), and scale length (L) (Fig. 1b), for assessing stock-related differences among species. The following morphometric indices were subsequently calculated and evaluated: R1/W, W/L, W/L1, W/L2, L1/L, L2/L, and L1/L2. To assess variation associated with the lateral line canal, scales from the remaining regions were analyzed.

### Scale landmark coordinations

In order to distinguish between distinct fish stocks, scales collected from the region beneath the pectoral fin of the *Nemipterus* species under study were examined using geomorphometric techniques. Eight homologous landmarks were digitized on consistent anatomical positions of each scale using tpsUtil and tpsDig2 software^44^. These landmarks included the ventro- and dorso-lateral tips of the rostral margin (landmarks 1 and 2), the dorso- and ventro-rostral centers (landmarks 3 and 7), the dorsal and ventral tips of separation line (landmarks 4 and 6), the posterior tip of the scale (landmark 5) and the focal point of the scale (landmark 8) (Fig. 1b).

### Microscopic study

Scales from the first four body regions were photographed using a stereomicroscope equipped with an AxioCam ERc 5s camera (Carl Zeiss–Promenade 10; 07745 Jena, Germany) and operated with Zeiss imaging software at the National Institute of Oceanography and Fisheries. Scales from all regions were subsequently examined using scanning electron microscopy (SEM) to assess their microstructural characteristics. For SEM preparation, cleaned and dried scales were mounted on specimen stubs using adhesive tape and coated with a 30-nm layer of gold. Electron micrographs were obtained using a JEOL JSM-5400LV SEM operated in backscattered electron mode at an accelerating voltage of 15 kV at the Assiut University Electron Microscope Center, Assiut, Egypt. Interpretation of SEM features followed the descriptions and criteria outlined in Hassanien^45^, Sadeghi et al.^13^, Al Jufaili et al.^1^, and Mekkawy, et al.^3^, the SEM results were interpreted and described.

### Statistical analysis

IBM SPSS version 26^46^ was used to compute descriptive statistics of the scale morphometric indices. A two-way analysis of variance (ANOVA; design: species + scale location + species × scale location) was performed to evaluate the effects of species and scale location. Post hoc comparisons were conducted using Tukey’s HSD test to assess interspecific variation among *Nemipterus* species. A multivariate analysis of variance (MANOVA) was also applied. In terms of homogeneity of variances, Levene’s test revealed significant findings for every variable.

A two-way permutational multivariate analysis of variance (PERMANOVA) was conducted using the PAST version 4.11 software^47^ to further examine interspecific variation based on multivariate distances (Euclidean Index, 9,999 permutations). Furthermore, principal component analysis (PCA), discriminant function analysis (DFA), and cluster analysis based on Mahalanobis distances were performed Classification accuracy in DFA was assessed using leave-one-out cross-validation. Frequency distributions of radii counts across species and sampling locations were compared using Chi-square tests (Pearson Chi-square and likelihood ratio) to detect significant intra- and interspecific differences among *Nemipterus* stocks.

Geometric morphometric analyses were conducted using MORPHOJ version 1.07c^48^. Generalized Procrustes Analysis (GPA) was used to standardize landmark configurations by removing variation due to size, position, and orientation, generating a set of shape variables^49^. Multivariate analyses, including PCA and Canonical Variate Analysis (CVA), were performed to examine interspecific differences in shape. Deformed outline drawings were generated to depict the shape variations identified by the CVA, using the mean shape as a reference framework for comparative analysis. The Mahalanobis distance (*P* < 0.0001) were used to evaluate the significance of mean shape differences among species. To assess the effects of size and shape variation in fish scales, distinct multivariate regression analyses of Procrustes coordinates on centroid size were conducted following Cavalcanti, et al.^50^.

The landmark coordinates for the three *Nemipterus* species were transformed into distance measurements using PAST software. Stock discrimination was further investigated using 28 landmark-transformed distances analyzed through DFA and Multivariate Analysis of Covariance (MANCOVA). These analyses were conducted using raw data, distance indices standardized by standard length (SL), and measurements corrected for Burnaby’s allometry and isometry, employing both PAST and SPSS packages.

## Results

### Overall structural characteristics of scales

**Fig. 2:**
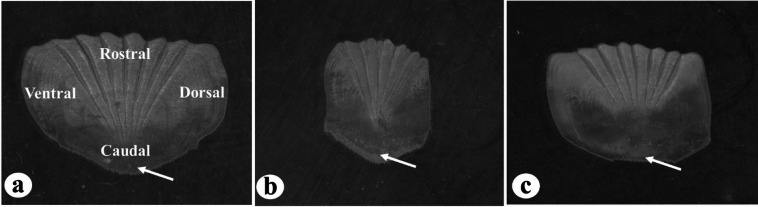
Types of scales observed in *N. japonicus*, representative for the other *Nemipterus* species studied. (**a**) scales broad at dorsoventral axis, (**b**) scales broad at anteroposterior axis (**c**) regenerated scales. Arrows indicate granulation segments.

The scales of the studied species are predominantly ctenoid with a sectioned structure, characterized by well-defined radii. Simple scales, characterized by the absence or faint development of radii, were not observed. The scales exhibited variation in shape they were predominantly polygonal (pentagonal or hexagonal) or rectangular in form. Among the examined body regions, the largest scales were observed in the post-opercular (POS) region. Scales collected from the first three body regions were generally broad along the dorsoventral axis (Fig. 2a), whereas those from the caudal peduncle (CPS) were broader along the anteroposterior axis (Fig. 2b). Each scale comprised four distinct fields: dorsal, ventral, rostral (anterior), and caudal (posterior).

Surface ornamentation across the scale fields was primarily characterized by circular patterns created by alternating ridges (circuli) intersected by distinct, deep, and narrow grooves known as radii, that extended radially from the focus toward the anterior margin. Depending on their position and point of termination, the radii were classified into two categories: primary and secondary (Fig. 1b). This configuration was clearly visible in most fields, except in the caudal field, where circuli were absent and replaced by granulated structures (Fig. 2). The scale focus was situated posteriorly. The rostral margin of the scales exhibited a wavy and striated appearance, contrasting with the crescent-shaped contour of the caudal margin. The dorsal and ventral fields consistently displayed a convex form across all examined body regions. Regenerated scales were also observed in various body regions, distinguished by the lack of surface ornamentation, especially in the central portion of the scale (Fig. 2c).

### Traditional morphometrics and meristics of scale

The number of R1 varied across different body regions, ranging from 4 to 10 in *N. zysron* and from 4 to 9 in both *N. randalli* and *N. japonicus*. In contrast, R2 counts ranged from 0 to 3 in *N. zysron*, 0 to 4 in *N. randalli*, and 0 to 1 in *N. japonicus* (Tables 1 and 2). A statistically significant correlation was observed between the number of R1 and body size in *N. zysron* (*P* < 0.01), suggesting that R1 development in this species is size-dependent. However, no significant correlation was observed between R1 count and body size in *N. randalli* and *N. japonicus*, indicating that this trait is not influenced by body size in these species. Conversely, a significant correlation between R2 count and fish size was found in *N. japonicus*, whereas no such relationship was detected in *N. zysron* or *N. randalli* (*P* > 0.01), implying that R2 count is independent of body size in the latter two species.

Descriptive statistics (mean ± SD) and occurrence percentages of R1 and R2 counts across the first four body regions of the examined *Nemipterus* species are summarized in Tables 1 and 2. The distribution of R1 differed significantly among body regions in all species (*P* < 0.001) and exhibited interspecific variation within each region (*P* < 0.035). The distribution of R2 varied significantly among body regions in *N. zysron* and *N. randalli* (*P* < 0.001), but not in *N. japonicus* (*P* > 0.206). Interspecific differences in R2 distribution were also detected across all examined regions (*P* < 0.033).

**Table 1:** Basic statistics (Mean ± SD) and percentage of occurrence of primary radii (R1) counts of the scales of the studied *Nemipterus* species.

Species	Regions	N	R1							Mean ± SD
			4	5	6	7	8	9	10	
*N. zysron*	BDFS	100	0	8	38	39	14	1	0	6.62 ± 0.86
	POS	56	1.79	8.93	26.79	33.93	19.64	7.14	1.79	6.89 ± 1.2
	BLLS	53	5.66	33.96	45.28	15.09	0	0	0	5.7 ± 0.8
	CPS	50	4	6	36	36	16	2	0	6.6 ± 1.03
*N. randalli*	BDFS	50	20	46	24	10	0	0	0	5.24 ± 0.9
	POS	41	2.44	19.51	19.51	39.02	12.20	7.32	0	6.61 ± 1.22
	BLLS	51	21.57	56.86	21.57	0	0	0	0	5 ± 0.66
	CPS	40	17.5	35	32.5	12.5	2.5	0	0	5.48 ± 1.01
*N. japonicus*	BDFS	40	7.5	35	37.5	20	0	0	0	5.7 ± 0.9
	POS	40	2.5	2.5	10	27.5	35	22.5	0	7.58 ± 1.17
	BLLS	40	5	30	57.5	5	2.5	0	0	5.7 ± 0.76
	CPS	40	12.5	45	40	2.5	0	0	0	5.33 ± 0.73

No. of scales (N), Beneath dorsal-fin scales (BDFS), post-operculum scales (POS), and beneath the lateral line scales (BLLS), caudal peduncle scales (CPS).

**Table 2:** Basic statistics (Mean ± SD) and percentage of occurrence of secondary radii (R2) counts of the scales of the studied *Nemipterus* species.

Species	Regions	N	R2					Mean ± SD
			0	1	2	3	4	
*N. zysron*	BDFS	100	99	1	0	0	0	0.01 ± 0.1
	POS	56	66.07	23.21	8.93	1.79	0	0.46 ± 0.74
	BLLS	53	84.91	15.09	0	0	0	0.15 ± 0.36
	CPS	50	100	0	0	0	0	0.00 ± 0.00
*N. randalli*	BDFS	50	80	16	4	0	0	0.24 ± 0.51
	POS	41	24.39	43.90	21.95	7.32	2.44	1.2 ± 0.98
	BLLS	51	92.16	7.84	0	0	0	0.1 ± 0.3
	CPS	40	100	0	0	0	0	0.00 ± 0.00
*N. japonicus*	BDFS	40	97.5	2.5	0	0	0	0.03 ± 0.16
	POS	40	95	5	0	0	0	0.05 ± 0.22
	BLLS	40	100	0	0	0	0	0.00 ± 0.00
	CPS	40	100	0	0	0	0	0.00 ± 0.00

No. of scales (N), Beneath dorsal-fin scales (BDFS), post-operculum scales (POS), and beneath the lateral line scales (BLLS), caudal peduncle scales (CPS).

The basic statistical values of scale morphometric indices obtained from the first four body regions are shown in Table 3. ANOVA revealed that species, body region, and their interaction significantly influenced all scale morphometric indices (*P* < 0.001). The parameters W/L2, L1/L and L1/L2, in region BDFS, L2/L in region POS, L1/L, L1/L2, W/L, W/L1, and W/L2 in region BLLS, as well as W/L, W/L1, and W/L2 in region CPS exhibited the highest values for *N. zysron*. The greatest values for *N. randalli* were observed in region BDFS for W/L, W/L1 and L2/L; in region POS for W/L, W/L1, W/L2, L1/L and L1/L2; and in regions BLLS and CPS for L2/L. The highest values for *N. japonicus* were observed in W/L in region BDFS, L1/L and L1/L2 in region CPS, and R1/W across all regions.

All body regions under examination showed interspecific differences in scale indices, with the exception of the BDFS region with regard to the W/L2 index. Moreover, intraspecific variations in scale indices were observed in all species examined. The PERMANOVA results on the traditional scale indices across different body regions of the three *Nemipterus* species indicated no significant effect of species (*P* > 0.01), whereas scale location and the interaction between species and scale location exhibited highly significant effects (*P* < 0.0001).

**Table 3:** Descriptive statistics, mean ± standard deviation (SD), range and number of scales (N) of the scale’s morphometric indices from the first four body regions of the studied *Nemipterus* species.

	Species	Locations			
		(Mean ± SD) (Min-Max) (N)	(Mean ± SD) (Min-Max) (N)	(Mean ± SD) (Min-Max) (N)	(Mean ± SD) (Min-Max) (N)
		BDFS	POS	BLLS	CPS
L1/L	*N. zysron*	74.83C3.67^Aa^ (65.96–82.5) (100)	78.31 ± 4.19^Ab^ (66.15–86.21) (56)	81.03 ± 2.64^Ac^ (73.81–87.23) (53)	79.35 ± 2.69^Ab^ (75-85.11) (50)
	*N. randalli*	72.58 ± 3.25^Ba^ (64-79.49) (50)	81.59 ± 2.88^Bb^ (73.08–85.48) (41)	78.82 ± 1.79^Bc^ (72.55-82) (51)	76.62 ± 2.66^Bd^ (70.45–81.4) (40)
	*N. japonicus*	73.63 ± 2.51A^Ba^ (68.57–78.72) (40)	80.15 ± 4.23^Bb^ (67.39–86.76) (40)	80.15 ± 2.31^Ab^ (76.32–86.05) (40)	80.49 ± 2.2^Ab^ (75-84.85) (40)
L2/L	*N. zysron*	24.73 ± 3.83^Aa^ (16.67–34.04) (100)	21.24 ± 4.31^Ab^ (13.8–35.3) (56)	18.24 ± 2.74^Ac^ (12.28–26.19) (53)	20.16 ± 2.93^Ab^ (12.12-25) (50)
	*N. randalli*	26.96 ± 3.27^Ba^ (20.51-34) (50)	17.97 ± 2.95^Bb^ (14.29–26.92) (41)	20.86 ± 2.04^Bc^ (16.67–27.45) (51)	22.99 ± 2.63^Bd^ (17.14–29.55) (40)
	*N. japonicus*	26.43 ± 2.6^Ba^ (20.41–33.33) (40)	19.85 ± 3.96^Ab^ (13.51–32.61) (40)	20.09 ± 2.18^Bb^ (14.63–24.32) (40)	20.02 ± 2.1^Ab^ (15.63-25) (40)
L1/L2	*N. zysron*	312.23 ± 64.19^Aa^ (193.75-483.33) (100)	386.51 ± 93.57^Ab^ (188.89–625) (56)	456.38 ± 86^Ac^ (281.82–700) (53)	404.93 ± 81.41^Ab^ (300–700) (50)
	*N. randalli*	274.33 ± 44.01^Ba^ (188.24–387.5) (50)	466.77 ± 80.95^Bb^ (271.43-588.89) (41)	381.91 ± 44.52^Bc^ (264.29–490) (51)	338.85 ± 51.35^Bd^ (238.46-466.67) (40)
	*N. japonicus*	282 ± 36.52^Ba^ (211.11–380) (40)	422.91 ± 101.91^Ab^ (206.67–640) (40)	405.21 ± 59.7^Bb^ (322.22-583.33) (40)	406.92 ± 50.01^Bb^ (300–520) (40)
W/L	*N. zysron*	119 ± 7.8^Aa^ (100-135.42) (100)	157.96 ± 9.92^Ab^ (136-180.65) (56)	121.29 ± 10.62^Aa^ (93.62-150.79) (53)	98.16 ± 6.64^Ac^ (84.44-113.33) (50)
	*N. randalli*	128.7 ± 10.19^Ba^ (102.27-144.74) (50)	175.62 ± 10.31^Bb^ (150.91-191.94) (41)	110.14 ± 12.9^Bc^ (77.59-131.75) (51)	92.72 ± 6.92^Bd^ (77.08-108.11) (40)
	*N. japonicus*	125.79 ± 7.9^Ba^ (112.77–140) (40)	152.5 ± 11.07^Cb^ (129.27-174.14) (40)	115.4 ± 12.58^Bc^ (89.74-137.21) (40)	88.26 ± 8.48^Cd^ (70-103.13) (40)
W/L1	*N. zysron*	159.34 ± 12.41^Aa^ (131.25–200) (100)	202.22 ± 15.9^Ab^ (171.88-246.51) (56)	149.84 ± 14.22^Ac^ (121.95-193.88) (53)	123.83 ± 9.39^Ad^ (108.57-145.45) (50)
	*N. randalli*	177.64 ± 15.84^Ba^ (136.36–206.9) (50)	215.36 ± 12.68^Bb^ (184.44–238) (41)	139.82 ± 16.91^Bc^ (102.27-170.59) (51)	121.17 ± 10.23^Ad^ (102.78-142.86) (40)
	*N. japonicus*	171.05 ± 12.53^Ca^ (143.24-194.44) (40)	190.72 ± 16.31^Cb^ (157.5-223.33) (40)	143.94 ± 14.98^ABc^ (112.9-166.67) (40)	109.71 ± 10.75^Bd^ (87.5-126.92) (40)
W/L2	*N. zysron*	493.54 ± 88.9^Aa^ (321.05–750) (100)	773.06 ± 160.79^Ab^ (394.44-1144.44) (56)	679.4 ± 116.66^Ac^ (440-1085.71) (53)	498.64 ± 88.98^Aa^ (362.5–775) (50)
	*N. randalli*	483.79 ± 68.19^Aa^ (370.59–687.5) (50)	1002.8 ± 170.9^Bb^ (621.43-1288.89) (41)	532.19 ± 76.14^Ba^ (346.15–680) (51)	408.79 ± 58.41^Bc^ (307.69-557.14) (40)
	*N. japonicus*	480.25 ± 55.13^Aa^ (388.89–640) (40)	798.38 ± 168.23^Ab^ (420-1155.56) (40)	583.93 ± 108.64^Cc^ (411.11-842.86) (40)	446.25 ± 69.2^Ca^ (300-583.33) (40)
R1/W	*N. zysron*	12.43 ± 2.87^Aa^ (7.58-20.00) (100)	8.23 ± 1.88^Ab^ (5.33–12.86) (56)	8.83 ± 2.37^Ab^ (5.33–14.89) (53)	17.97 ± 4.84^Ac^ (8.89–31.03) (50)
	*N. randalli*	9.37 ± 1.62^Ba^ (6.06–13.64) (50)	6.78 ± 1.31^Bb^ (4.42–9.09) (41)	7.901 ± 1.29^Bc^ (5.71–11.11) (51)	15.1 ± 2.82^Bd^ (9.52–21.62) (40)
	*N. japonicus*	12.65 ± 2.57^Aa^ (6.41–17.65) (40)	11.22 ± 3.29^Ca^ (5.31–16.98) (40)	12.25 ± 2.18^Ca^ (8.51–17.65) (40)	18.8 ± 3.64^Ab^ (11.43–26.09) (40)

Similar uppercase letters vertically indicate no significant differences between species at *P* < 0.05.

Similar lowercase letters horizontally indicate no significant differences between locations at *P* < 0.05.

**Fig. 3:**
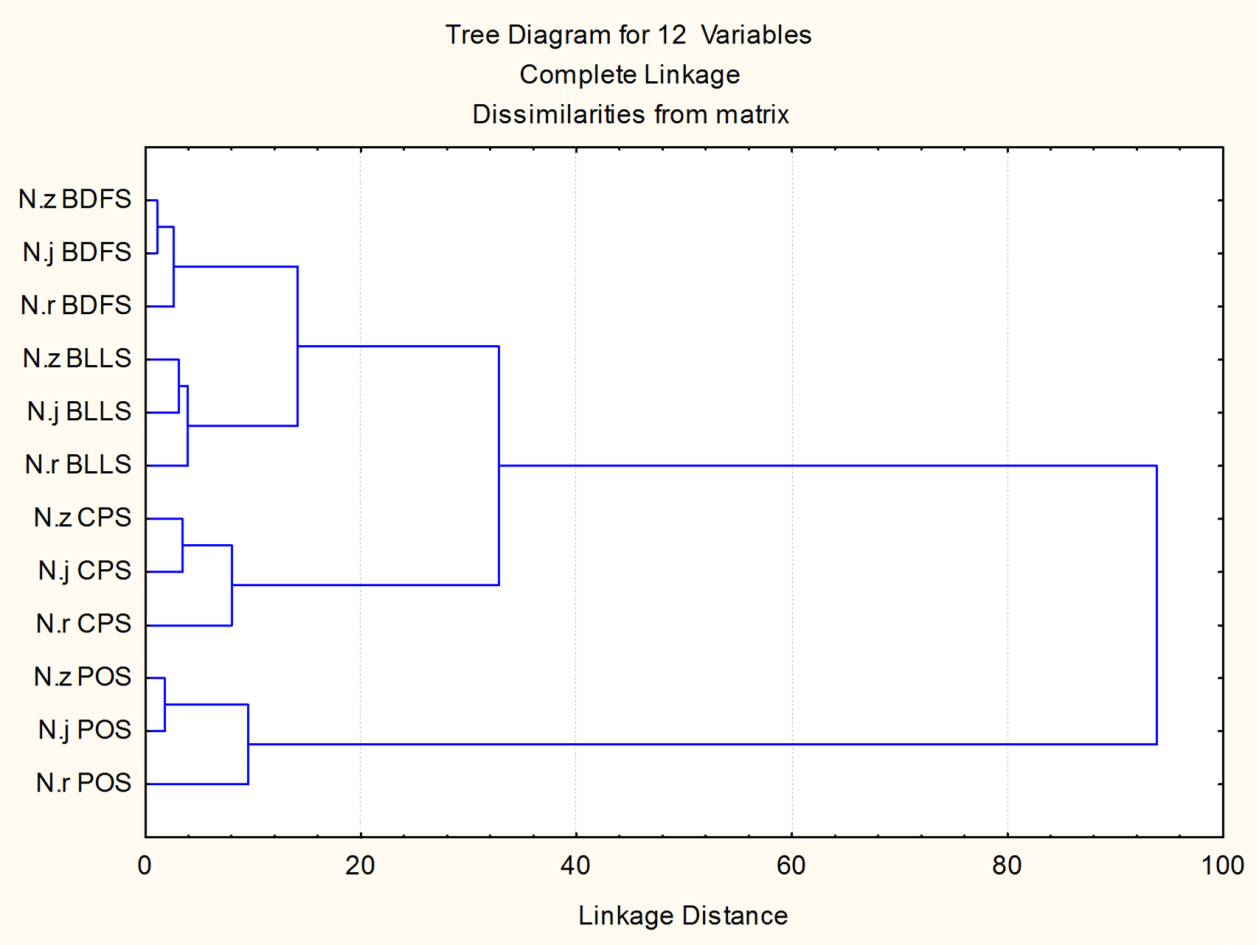
Clustering of the studied *Nemipterus* species examined, utilizing squared Mahalanobis distances. The species and region abbreviations are displayed alongside the scale morphometric indices on the Y-axis. (N.z: *N. zysron*, N.r: *N. randalli*, N.j: *N. japonicus*)

Figure 3 illustrates the clustering of scale locations derived from the significant Mahalanobis distance matrix (*P* < 0.0001) of the examined scale indices. No distinct clustering pattern was observed in relation to species or body regions, as there was considerable overlap between the body regions of different species. Interspecific differences were evident, irrespective of scale location, as demonstrated by the Mahalanobis distances (*P* < 0.0001). *N. japonicus* formed a distinct cluster, whereas the remaining species showed no clear separation on the CVI (82.49%), with 50.9% of cross-validated grouped cases accurately classified.

### Geometric morphometric analysis

**Fig. 4:**
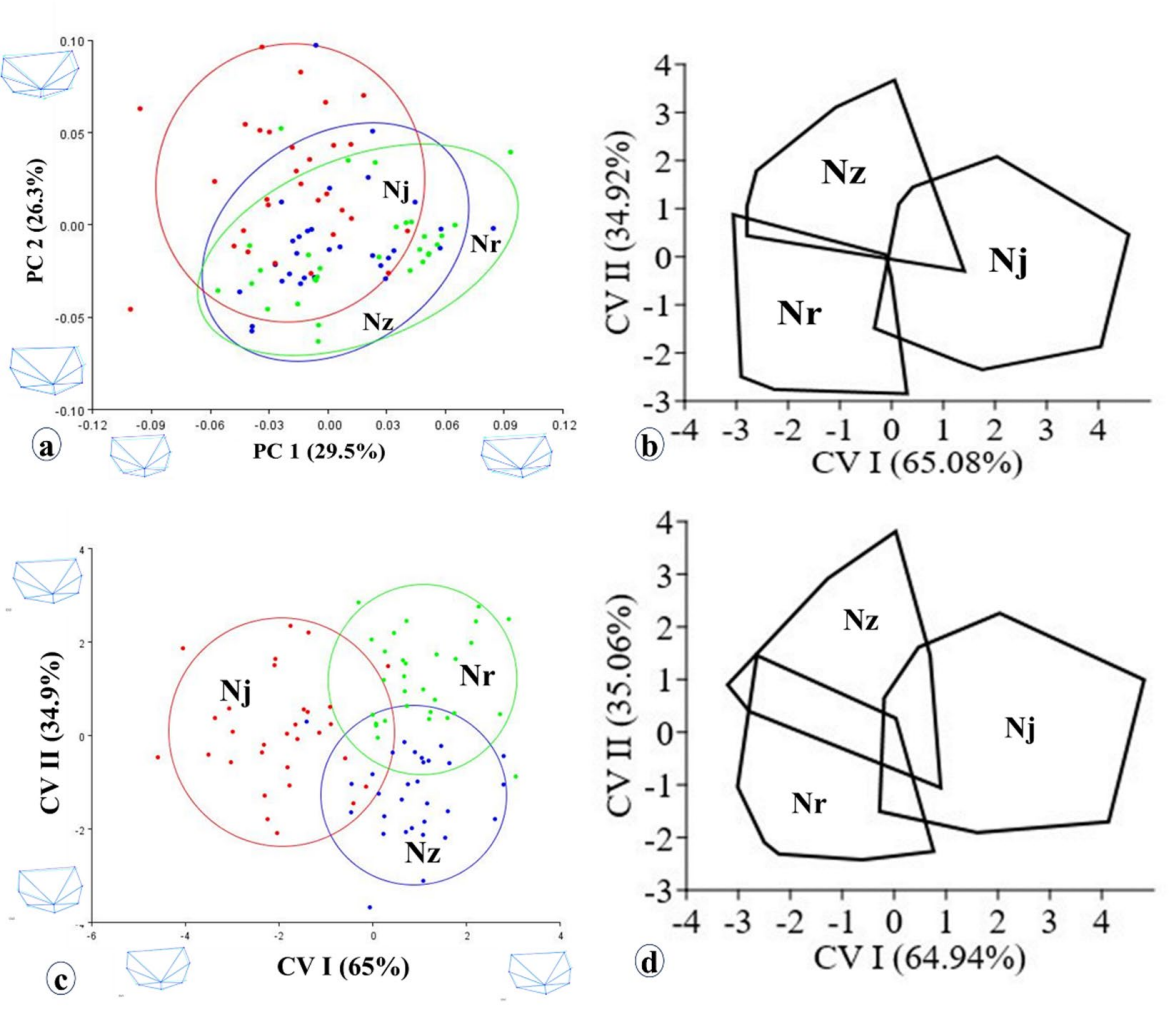
(**a**) Distribution of scores for the first two principal components (PC I, PC II) derived from the Procrustes coordinates of scales across the species under study. (**b**) Scatter plot from Canonical Variable Analysis (CVA) showing the scale shape based on coordinates (CVI, CVII). (**c**) CVA scatter plot illustrates the regression residuals of the Procrustes coordinates. (**d**) Discriminant function analysis of the CVA scatter plot based on the principal components of regression residuals derived from the Procrustes coordinates. Nz: *N. zysron*, Nr: *N. randalli*, Nj: *N. japonicus*.

Principal components analysis (PCA) of the Procrustes coordinates of scales located beneth the pectoral fin revealed interspecific variations among the *Nemipterus* species studied (Fig. 4a). Notably, *N. zysron* was positioned within the range of the other two overlapping species on PCI (29.5%). Canonical variate analysis (CVA) of these coordinates further demonstrated clearer separation of species with minimal interspecific overlap on CVI (65.08%) (Fig. 4b). The predicted group membership accuracy for the original cases of *N. zysron*, *N. randalli*, and *N. japonicus* was 96.7%, 93.3%, and 90.0%, respectively, with an overall mean accuracy of 93.3% for correctly classified original cases. In contrast, the average cross-validation accuracy for grouped cases was 80.0%, with correct classification rates of 76.7% for *N. zysron*, 83.3% for *N. randalli*, and 80.0% for *N. japonicus*. Furthermore, a statistically significant difference was observed among species based on the combined Procrustes coordinates after controlling for standard length (SL) (Wilks’ λ = 0.172, *p* < 0.0001, partial η² = 0.586).

The DFA of the regression residuals of the Procrustes coordinates revealed an interspecific pattern of variation consistent with the previous analysis (Fig. 4c). The predicted group membership accuracy for the original cases of *N. zysron*, *N. randalli*, and *N. japonicus* was 96.7%, 93.3%, and 90.0%, respectively, resulting in an average accuracy of 93.3% for correctly classified original cases. In contrast, the average accuracy for cross-validated grouped cases was also 93.3%, with correct classification rates of 96.7% for *N. zysron*, 93.3% for *N. randalli*, and 90.0% for *N. japonicus*. Furthermore, a statistically significant difference was found among species based on the combined regression residuals after controlling for centroid size (Wilks’ λ = 0.174, *p* < 0.0001, partial η² = 0.583).

The DFA of the principal components of regression residuals derived from the Procrustes coordinates exhibited an interspecific variation pattern that substantially coincided with the aforementioned patterns (Fig. 4d). The predicted group membership for the original cases of *N. zysron*, *N. randalli*, and *N. japonicus* is 96.7%, 93.3%, and 90.0%, respectively, resulting in a mean classification accuracy of 93.3% for the original grouped cases. An average of 81.1% of cross-validated grouped cases were correctly classified for *N. zysron* (80.0%), *N. randalli* (80.0%), and *N. japonicus* (83.3%). A statistically significant difference among species was observed in the combined principal components of regression residuals after adjusting for centroid size (Wilks’ A = 0.166, *p* < 0.0001, partial η2 = 0.592).

**Fig. 5:**
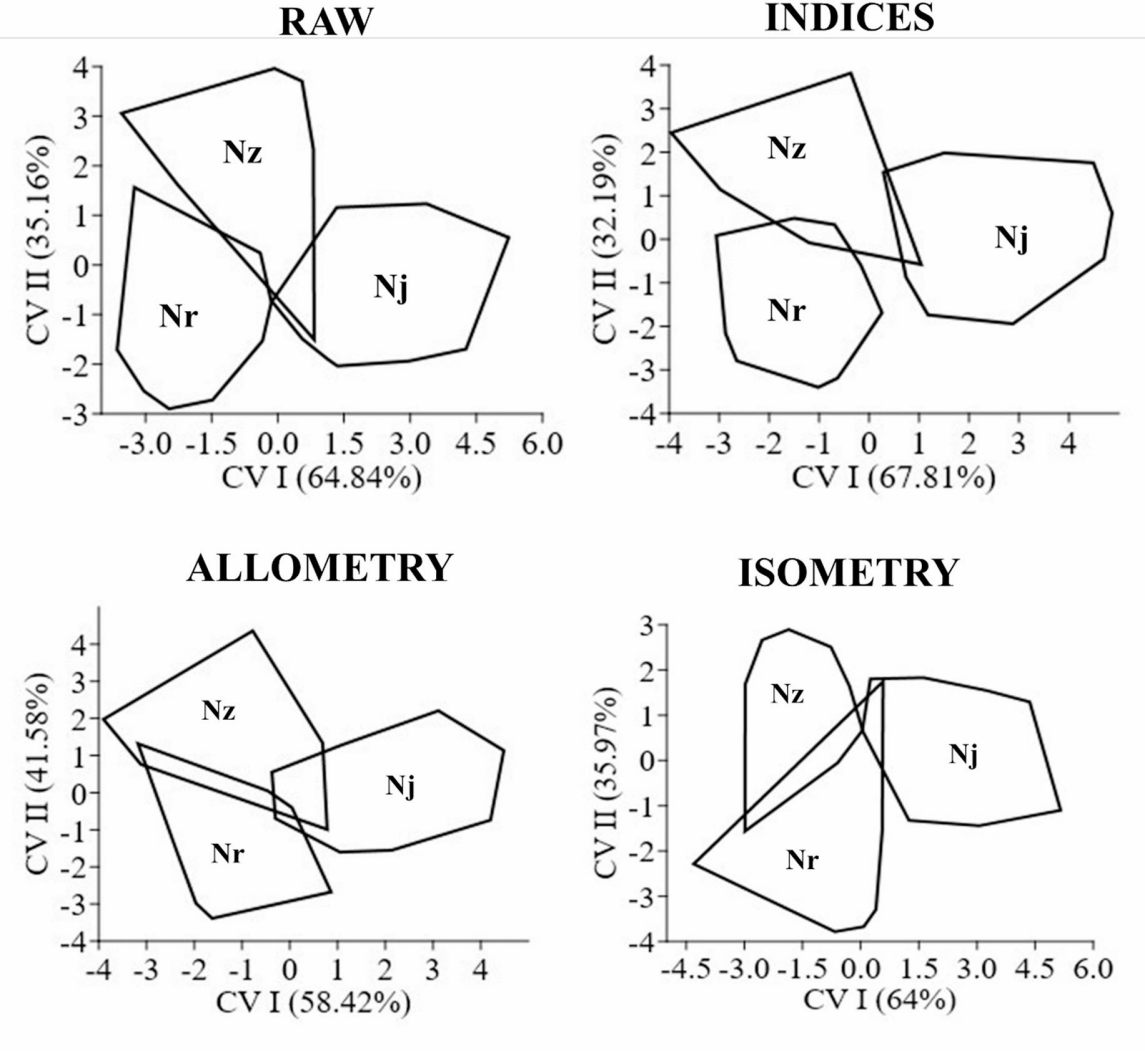
Scatter plot of Canonical Variable Analysis (CVA) depicting the landmarks-transformed distances of the species studied from Hurghada, Red Sea, Egypt, based on scale morphology. Nz: *N. zysron*, Nr: *N. randalli*, and Nj: *N. japonicus*.

The DFA of landmark-transformed distances revealed interspecific variation among the studied *Nemipterus* species. Raw landmark distances (without normalization) showed species separation with some overlap (CVI = 64.84%) (Fig. 5a). When indices were scaled relative to standard length (SL), species clusters separate more distinctly, especially between *N. japonicus* and the other two species (CVI = 67.81%) (Fig. 5b). After removing allometric effects, the species clusters remained partially overlapping; however, *N. japonicus* tended to form a distinct group. In contrast, controlling for isometric size scaling resulted in a clearer separation of *N. japonicus*, while *N. randalli* and *N. zysron* remained partially overlapping (Fig. 5c, d). Removing allometric effects further enhanced separation among species (Fig. 5c). In contrast, controlling for isometric size scaling (i.e., analyzing shape variation independent of overall size) resulted in minor overlap between *N. randalli* and *N. zysron*, while *N. japonicus* remained distinct from *N. zysron* (Fig. 5d). *Nenipterus* species exhibited average correct classification rates of 91.1%, 91.1%, 90%, and 92.2% for raw distance, indices, isometry, and allometry transformed distances, respectively. Conversely, the species exhibited means of 76.7%, 73.3%, 75.6%, and 76.7% for correctly classified cross-validated grouped cases based on raw distance, indices, isometry, and allometry transformed distances, respectively. Clustering utilizing Mahalanobis distance revealed the subsequent relationship patterns among *Nemipterus* species: *N. zysron* + *N. randalli* vs. *N. japonicus* for raw distance, distance indices and isometry and allometry transformed distances.

In conclusion, significant interspecific variations in size, shape, and shape-size among the studied *Nemipterus* species were observed through both univariate and multivariate analyses of their traditional and landmark-based scale morphometric traits. Isometry and allometry transformed distances, revealed unique patterns of shape variation.

### Scanning electron microscope studies (SEM)

#### Rostral field

##### The inter-radial tongues and the first circulus

**Fig. 6:**
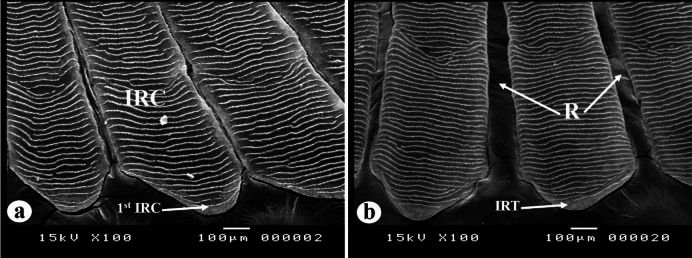
SEM of the rostral field of *N. japonicus* scales, representing the other two studied species, illustrating the radii (R), inter-radial tongues (IRT), inter-radial circuli (IRC) and the first inter-radial circulus (1st IRC). (**a**) Conical tongues with a crescent-shaped first inter-radial circulus. (**b**) Conical tongues with a straight first inter-radial circulus.

Within the inter-radial space, the rostral margins of the scales exhibit tongue-like projections that lack circuli near the rim (Fig. 6). Two distinct forms of these projections, along with the first inter-radial circulus, are observed in the scales of the examined nemipterid species. The first form is characterized by conical tongue-like projections and a crescent-shaped first inter-radial circulus, which was identified in *N. zysron* (BDFS, POS, CPS, MLLS, and PLLS), *N. randalli* (BDFS), and *N. japonicus* (BDFS) (Fig. 6a). The second form exhibits conical tongue-like projections with a straight first inter-radial circulus (Fig. 6b), this form was recorded in *N. zysron* (BLLS and ALLS), *N. randalli* and *N. japonicus* (all regions except BDFS).

##### Radial grooves

**Fig. 7:**
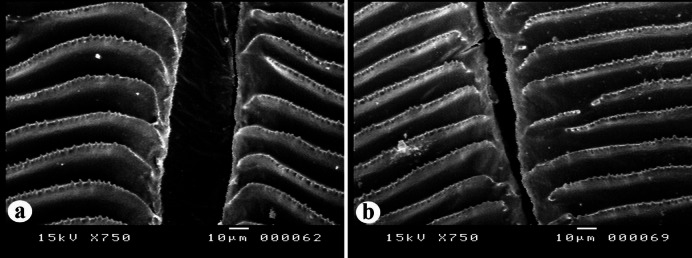
SEM of the radial grooves in the *Nemipterus* species under study. (**a**) Wide, deep grooves with a thin, membrane-like structure in *N. randalli* scales, representing the other two studied species. (**b**) Narrow, deep grooves with a thin, membrane-like structure in *N. randalli* scales, representing *N. zysron*.

The radial grooves of the scales of the nemipterid species under study are categorized into two distinct forms (Fig. 7): **Form 1**: wide and deep grooves with a thin membrane-like structure (Fig. 7a). This form was recorded in *N. zysron* (BDFS, BLLS & PLLS), *N. randalli* (BDFS, BLLS, ALLS, MLLS & PLLS) and *N. japonicus* (all regions). **Form 2**: narrow and deep grooves with a thin membrane-like structure (Fig. 7b). This form was recorded in *N. zysron* (POS, CPS, ALLS & MLLS) and *N. randalli* (POS & CPS). In both types, the ridges extend almost to the edge of the groove.

##### The inter-radial circuli, grooves and denticles

**Fig. 8:**
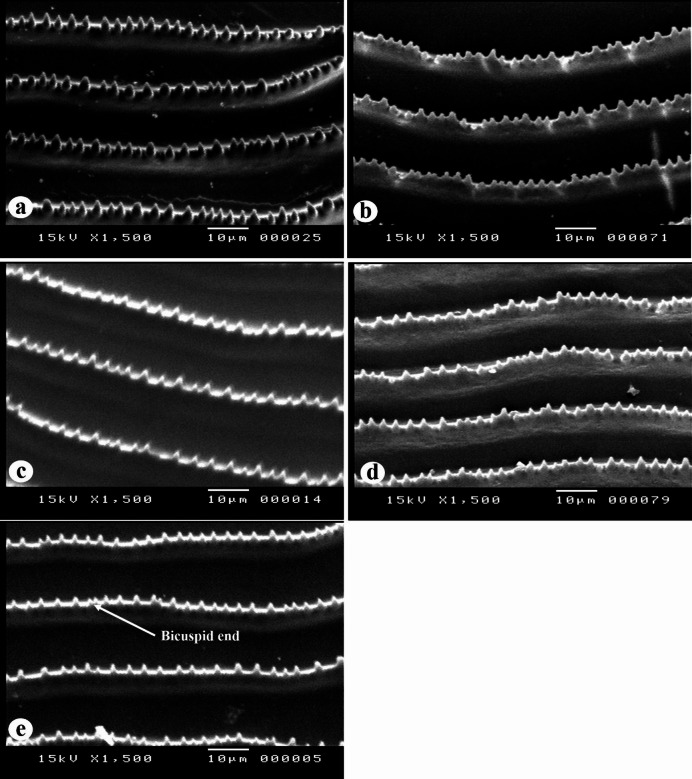
SEM of the inter-radial circuli, grooves and denticles of the three studied *Nemipterus* species; (**a**) conical denticles, featuring recurved, hook-like teeth in *N. zysron* (**b**) blunt denticles on *N. randalli* circuli as representative of *N. zysron*, (**c**) conical denticles with unicuspid end on *N. japonicus* circuli representing the other two species showing, (**d**) irregular denticles in *N. randalli*, (e) conical denticles with unicuspid and bicuspid ends in *N. japonicus.*

The inter-circular grooves in the rostral field were narrow and deep. The circuli exhibited small denticles within the inter-radial space. Five distinct forms of denticles were recorded on the dorsal surfaces of the inter-radial circuli (Fig. 8): **Form 1**: The denticles are conical in shape, featuring recurved, hook-like teeth (Fig. 8a) recorded in *N. zysron* (BDFS, BLLS & CPS). **Form 2**: blunt denticles are (Fig. 8b) recorded in *N. zysron* (POS) and *N. randalli* (BDFS). **Form 3**: conical denticles are with unicuspid ends (Fig. 8c) recorded in *N. zysron* (ALLS, MLLS & PLLS), *N. randalli* (POS, ALLS, MLLS & PLLS) and *N. japonicus* (POS, CPS ALLS, MLLS & PLLS). **Form 4**: the denticles are irregular shape (Fig. 8d) recorded in *N. randalli* (CPS & BLLS). **Form 5**: the denticles are conical with bicuspid and unicuspid ends (Fig. 8e) recorded in *N. japonicus* (BDFS & BLLS).

##### Outer lateral circuli, grooves and denticles

**Fig. 9:**
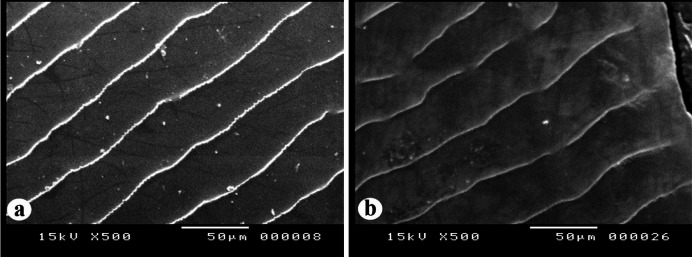
SEM of the outer lateral circuli, grooves and denticles of the studied *Nemipterus* species; (**a**) The outer circuli are thin and bear denticles with flat and wide grooves in *N. zysron* as representative of *N. randalli*; (**b**) The outer circuli are thin and free of denticles with flat and wide grooves in *N. japonicus* representing the other two species.

In the studied *Nemipterus* species, the outermost lateral circuli display thin, flat, and wide grooves. These circuli either bear denticles (Fig. 9a), as observed in *N. zysron* (BDFS, POS, CPS, MLLS & PLLS) and *N. randalli* (BDFS, POS & BLLS), or are free of denticles (Fig. 9b) as documented in *N. zysron* (BLLS & ALLS), *N. randalli* (CPS, ALLS, MLLS & PLLS), and *N. japonicus* (all regions).

##### Inner lateral circuli, grooves and denticles

**Fig. 10:**
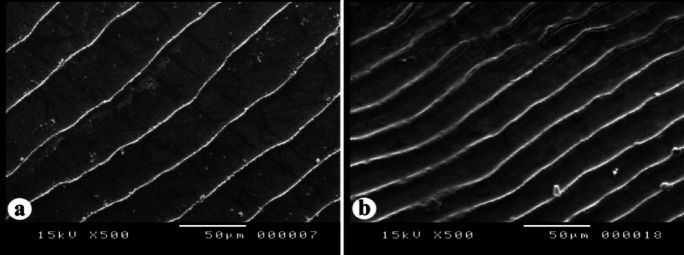
SEM of the inner lateral circuli, grooves and denticles of the studied *Nemipterus* species; (**a**) the inner circuli are thin, bear many denticles with flat, shallow and wide grooves in *N. zysron* representing the other two species; (**b**) The inner circuli are thick, bear few denticles with flat deeper and narrower grooves in *N. japonicus*.

In the three studied *Nemipterus* species, the inner lateral circuli display two types: either thin, bearing numerous denticles with flat, shallow, and wide grooves (Fig. 10a), observed in *N. zysron* and *N. randalli* (all regions) and *N. japonicus* (BDFS, BLLS, CPS, ALLS, MLLS & PLLS), or thick, bearing few denticles with flat, deeper, and narrower grooves (Fig. 10b), documented in *N. japonicus* (POS).

#### The focus region

**Fig. 11:**
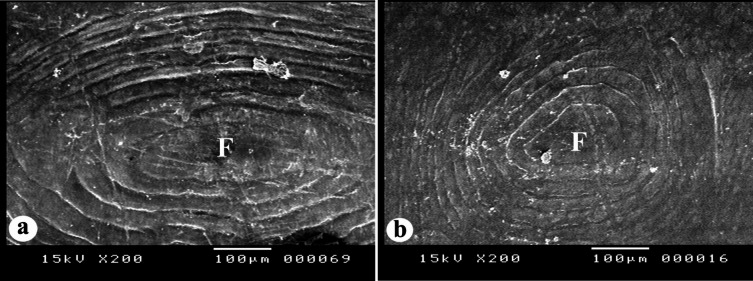
SEM of the focus region in the three studied *Nemipterus* species; (**a**) oval shaped focus in *N. japonicus* as representative of *N. randalli*, (**b**) round shaped focus in *N. zysron* representing the other two species.

The focus region in the three *Nemipterus* species appears in two forms: it is either oval-shaped and surrounded by oval ridges (circuli), as observed in *N. randalli* (BDFS, POS & BLLS) and *N. japonicus* (POS & BLLS) (Fig. 11a) or round-shaped and surrounded by round ridges, as documented in *N. zysron* (all regions), *N. randalli* (CPS) and *N. japonicus* (BDFS & CPS) (Fig. 11b).

#### Caudal field

**Fig. 12:**
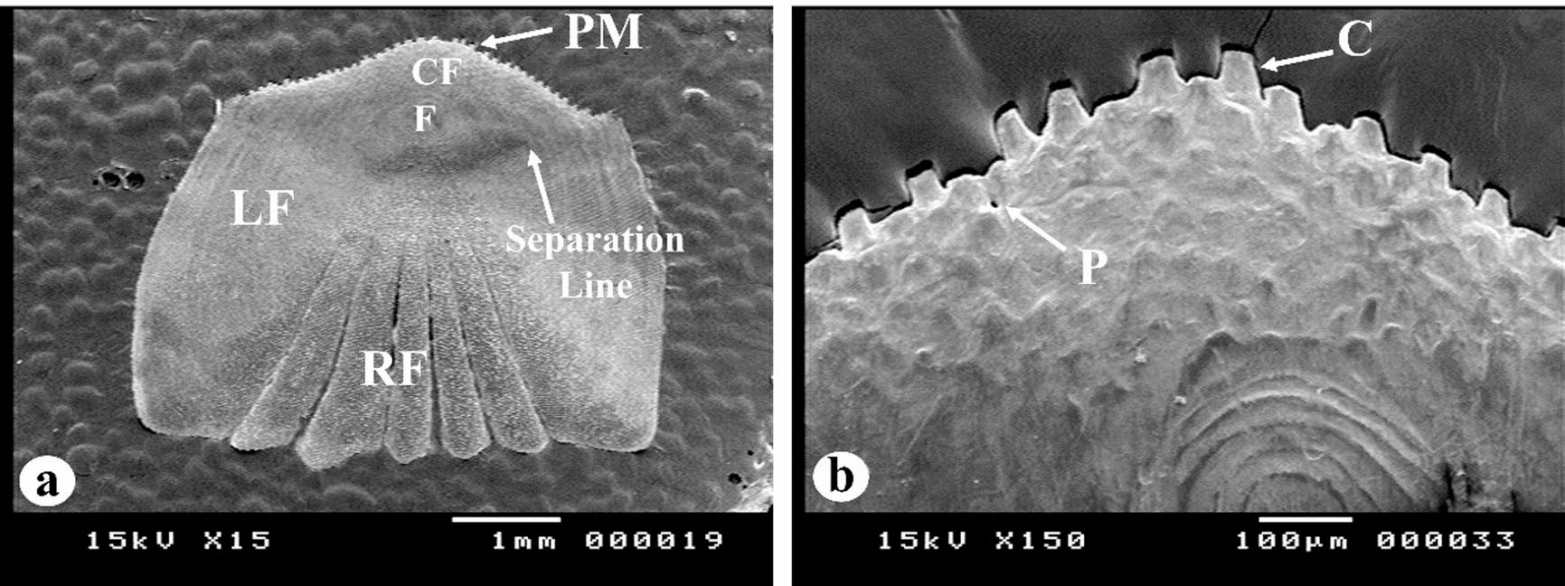
SEM of the caudal region of *N. japonicus* scales, representing the other two species. The caudal region has fine segment with pores (P), ctenii (C), focus (F), posterior margin (PM), rostral field (RF), caudal field (CF), lateral field (LF).

In all the studied species, the caudal field area is weakly delineated from the anterior rostral field by the separation line, and the posterior rim exhibits a curved shape, featuring ctenii (Fig. 12a). The granulation area in the caudal field is devoid of circuli; yet, it features fine segments and is permeated by many pores throughout all regions (Fig. 12b).

**Fig. 13:**
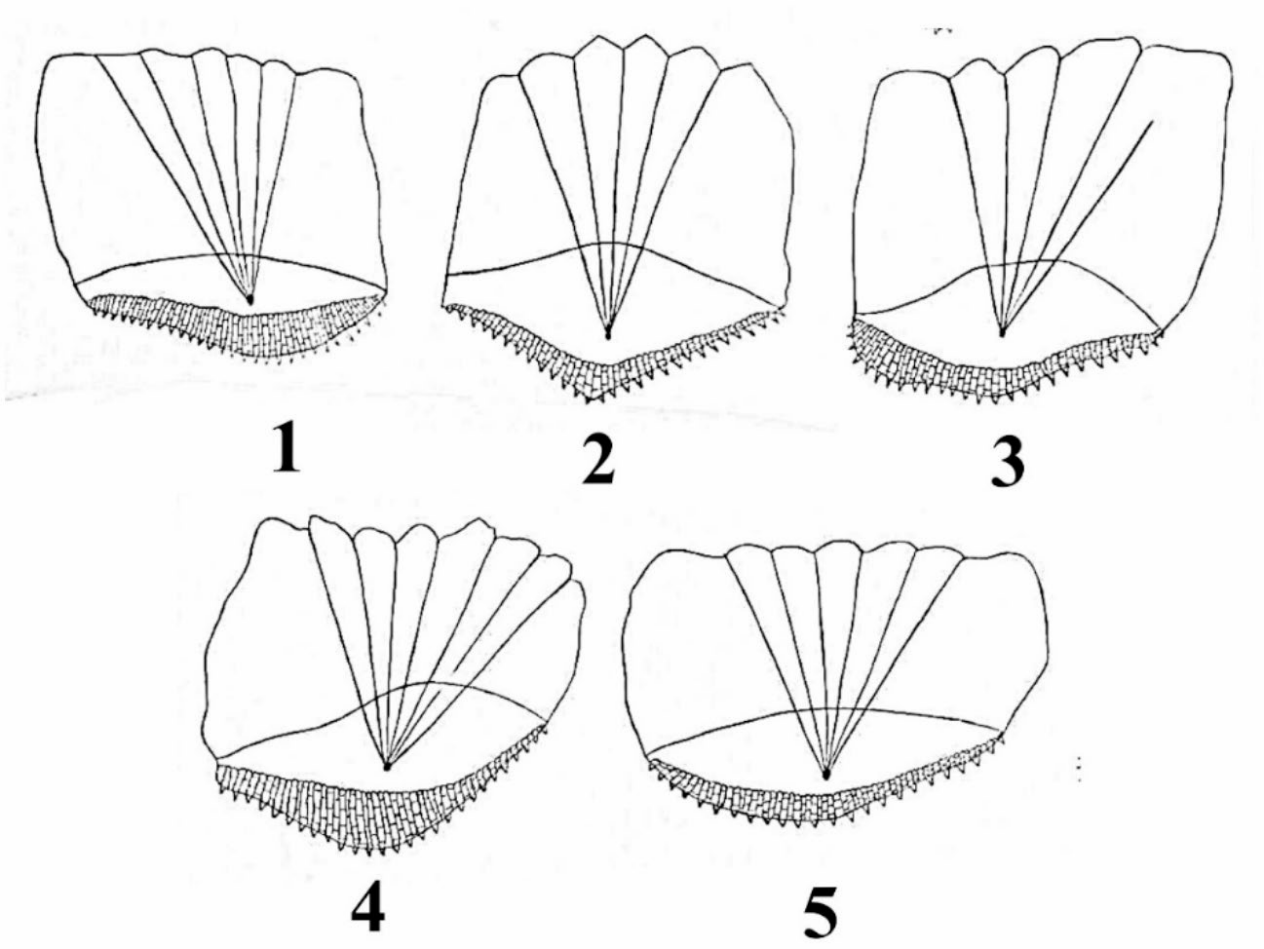
Schematic diagrams illustrating the forms of scales identified in the studied *Nemipterus* species according to the separation line, the posterior rim and the shape of granulation area in the caudal field.

Based on the separation line, the shape of the caudal region and the posterior rim five distinct scale forms were identified in the three *Nemipterus* species studied (Fig. 13): **Form 1**: The separation line is almost straight and the posterior rim is irregular crescent resulting in an oval caudal area. **Form 2**: The separation line is convex and the posterior rim is V-shaped resulting in a nearly conical caudal area. **Form 3**: The separation line and the posterior rim are irregular resulting in an elongated irregular oval caudal area. **Form 4**: The separation line is irregular crescent and the posterior rim is rounded resulting in almost a parallelogram caudal area. **Form 5**: The separation line is almost straight and the posterior rim is rounded resulting in an oval caudal area.

#### Lateral line scales

**Fig. 14:**
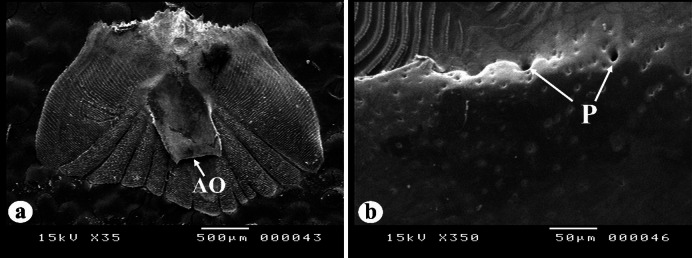
SEM of *N. japonicus* scales, representing the other two species showing the lateral line canal characteristics including anterior opening (AO) and pores (P).

In the studied species, the lateral line scales generally feature a wide tube (canal) that runs parallel to the anteroposterior axis of the scale in a straight manner, while some other tubes are oriented obliquely to this axis (Fig. 14a). This canal exhibits a posterior opening positioned medially within the focus region on the inner surface of the scale and located at a considerable distance from the posterior margin. The anterior opening is similarly situated away from the anterior margin. Additionally, lateral line canal pores are present on both sides of the canal (Fig. 14b).

## Discussion

Scale morphometrics, geomorphometrics, and microstructural analyses have been widely utilized in comparative, phylogenetic, and stock identification studies of fish species^2,3,13,15,18,20,25,51,52^. The methodologies utilized by these authors often incorporate univariate and multivariate analyses^3,5,8,20^, scanning electron microscopy (SEM)^1,3,8,13,18^ and geomorphological analyses^2,16,17,20^.

To date, only a limited number of studies have investigated the scale morphology of nemipterid species, collectively demonstrating that scale characteristics can serve as reliable indicators for distinguishing among *Nemipterus* stocks. Renjith, et al.^51^ analyzed morphological variation in the tenth lateral line scale of *N. japonicus*, *N. bipunctatus* and *N. randalli* using elliptical Fourier analysis, revealing distinct interspecific differences in scale shape and highlighting the taxonomic value of scale morphology within the genus. Devi, et al.^7^ further described the scales of *N. japonicus* as exhibiting clear morphological variations across three different body regions, while Ujjania and Jaiswar^53^ compared variations in scale size between *N. japonicus*, *N. bipunctatus* and *N. randalli*, reinforcing the diagnostic potential of scale-based traits.

The predominant scale type among marine fishes is the elasmoid type, encompassing both cycloid and ctenoid scales^7^. In the present study, the scales of *Nemipterus* species were identified as ctenoid and sectioned in type, exhibiting distinct surface ornamentation. These observations corroborate the findings of Devi, et al.^7^, who characterized the scale morphology of several species including *N. japonicus*, and those of Lelli, et al.^41^, who examined *N. randalli* from the eastern Mediterranean Sea. In addition, our results are consistent with the general morphological patterns reported in various marine taxa^1,3,13,18^.

According to Spinner, et al.^54^, the evolution of ctenoid scales was likely driven by mechanical interactions with the surrounding environment, offering an adaptive advantage by reducing physical damage. This adaptation is particularly relevant for demersal species such as *Nemipterus*, which inhabit areas where contact with the sea bottom, rocks, and corals is frequent. Thus, the presence of ctenoid scales in *Nemipterus* may represent an evolutionary response to the mechanical constraints of a demersal lifestyle. Moreover, beyond their protective function, ctenoid scales also contribute to hydrodynamic efficiency. Complementary to this, Harabawy, et al.^11^ reported that the epidermal surface of fishes, especially those inhabiting coral reefs, is exposed to frictional forces generated by water movement. By regulating turbulence within the boundary layer, ctenoid scales help maintain smooth water flow along the body surface, thereby reducing drag and enhancing swimming performance^2^. Furthermore, some fish species exhibit both cycloid and ctenoid scales, reflecting variations in scale morphology and functional adaptations^9,13,15^.

Scale shape is also thought to influence swimming performance. For instance, Ibañez, et al.^55^ suggested that scales elongated along the anteroposterior axis may reduce thrust and water pressure generated during swimming. In contrast, scales that are comparatively wide along the dorsoventral axis and shorter in the anteroposterior axis are thought to be advantageous for a subcarangiform swimming mode. In the present study, scales of the latter form were predominantly observed in the first three body regions. However, due to the lack of detailed information regarding the swimming behavior and performance of nemipterid species, a potential association between scale shape and swimming mode cannot be formally evaluated. The caudal peduncle scales are clearly distinguishable from those of the other body regions with respect to their size (as indicated by the W/L ratio) and overall shape. The elongation of these scales along the anteroposterior axis suggests a functional role in enhancing swimming efficiency by modulating thrust and water pressure^55^. These findings are consistent with observations reported for cichlid species from Lake Tanganyika^2^.

The current study, along with various other investigations^3,12,45,56^ supports the validity of scale radii-meristics distribution and morphometrics in the identification of fish species and stocks. The number and morphology of radii serve as valuable taxonomic characters for species identification^1^. In the present study, both primary and secondary radii exhibited interspecific differences in the studied species. The primary radii varied intraspecifically among body regions in all species and was size-dependent only in *N. zysron*. Whereas, the secondary radii showed intraspecific variations in *N. zysron* and *N. randalli*, with size-dependence observed solely in *N. japonicus*. Few studies have investigated such variations^1,3,12,56,57^. In addition, primary radii in *Nemipterus* species exhibited higher counts than secondary radii, a situation also recorded previously by different authors^3,45,58^. According to Raffealla and Nath^59^ the formation of primary, secondary, and tertiary radii is generally considered a growth-related phenomenon.

Moreover, it was suggested that the radii exhibited a weak influence from genetic factors, suggesting that other environmental or physiological factors may play a more significant role. In this regard, the enhanced nutritional conditions of the fish may correlate with an increased number of radii^60^. Given that radii represent gaps in scale ossification that contribute to scale flexibility^61^ while maintaining protective functionality^62^, their variation could reflect adaptive responses to environmental conditions rather than strictly genetic determinants. However, due to the absence of data on body flexibility of *Nemipterus*, it is currently impossible to comment on such a relationship. In addition, Esmaeili and Gholami^61^ reported that the number of radii depends on the scale location on fish body. Our results revealed that the BDFS, POS, and CPS regions showed the greatest number of radii counts among the examined species. According to Wainwright and Lauder^62^, such elevated radii counts typically occur in body areas that are inherently curved, such as the dorsal region, or in those subjected to greater lateral bending during locomotion, such as the caudal peduncle and tail.

In the present study, both intraspecific and interspecific relationships were reflected through quantitative, size-independent morphometric indices of the scales. The obtained results are consistent with those of Ujjania and Jaiswar^53^, who investigated *N. japonicus* and *N. randalli*. Furthermore, several studies have employed comparable morphometric indices of fish scales in other teleost species^1,3,5,8,11,63,64^. The observed morphological differentiation among the studied species may be attributed to environmental, geographical, and biological variations influencing phenotypic expression^53^. In contrast to the present findings, previous authors did not address the variability of primary radii in relation to scale width across different species and sampling locations.

Numerous investigations have focused on the analysis of fish populations and species identification through the examination of scale geometry utilizing different scale landmarks in multivariate size and shape assessments^2–4,17,19,20^. Also, the geometric analysis is harmless, rapid, and cost-effective than genetic analysis^16^.

Our results revealed clear species-specific scale shape differences. This was supported by high classification accuracies and statistical significance, demonstrating that the methods effectively discriminate species by scale shape variation. In addition, landmarks transformed distances, substantially improved species discrimination. *N. japonicus* consistently clusters apart, whereas *N. zysron* and *N. randalli*, though more similar, are better separated once size-related shape variation is accounted for. This highlights the essential role of morphometric data transformations for accurate species classification, especially in closely related taxa with subtle morphological differences. These results align with recent studies employing geometric morphometrics for species discrimination in other fish taxa^2,17,20,65^.​.

Various studies have investigated the ultrastructure and surface ornamentation of scales in the rostral and caudal regions to distinguish between species^1,3,8,13,15^. In the present study, the initial inter-radial circulus exhibited either a crescentic or straight configuration, a feature that may represent species-specific variation. Similar observations have been documented in various fish taxa^3,11,45,56^. Correspondingly, Lippitsch^66^ noted that the morphology of the first inter-radial circuli is characteristic within cichlid species, although it may occasionally be influenced by environmental conditions. Furthermore, the shape of the first circuli has been recognized as a potentially valuable taxonomic character in certain groups, such as goatfishes (Mullidae)^15^.

Previous studies have reported that denticles exhibit considerable variation in shape and size across different fish species^1,3,8,15,18^. Variations in denticle morphology have been shown to depend on species identity, body size, and the specific location of the scales on the body^6,15,18^. The present investigation did not reveal significant interspecific differences in denticle morphology among the three *Nemipterus* species examined. This finding aligns with the observations of Al Jufaili, et al.^1^, who indicated that the taxonomic significance of denticles in *Garra shamal* remains uncertain, and with Gholami, et al.^67^, who reported that the microstructure of scales, particularly with respect to denticles, is insufficient for distinguishing among aphaniid species but may assist in differentiating certain populations. Conversely, Ferrito, et al.^68^ demonstrated that denticle morphology remains constant throughout the life of the fish and represents a reliable taxonomic feature for species identification. This is further supported by Lippitsch^66^ who reported that denticle morphology may serve as a useful character in phylogenetic analyses.

Moreover, the presence of denticles on the inter-radial circuli and rostrolateral regions of scales in *Nemipterus* species suggests a functional role in reducing frictional forces through mechanical anchoring, a mechanism previously described in several other fish taxa^12,69,70^. In addition, the circuli of the rostral field bear a higher density of denticles compared to those of the lateral fields, which likely enhances the attachment of scales to the underlying skin^9^. Yang, et al.^71^ proposed that the circuli and other microroughness features on the surface of fish scales serve to concentrate tensile stresses within the depressions between circuli during scale bending. By restricting tensile stress to these regions, the overall stress experienced during bending is reduced, thereby influencing the magnitude of force required to achieve a given level of deformation^62^.

The present study observed that the outer lateral circuli of *N. japonicus*, as well as the inner and outer lateral circuli of scales in specific locations of the other two *Nemipterus* species examined, lacked denticles. The absence of denticles in certain regions of the scales suggest that anchoring is not crucial in these regions, particularly in newly formed circuli^3^. These findings are consistent with the observations of Mekkawy, et al.^12^, Mekkawy, et al.^3^ and Mahmoud, et al.^72^.

In the studied nemipterid species, the focus exhibits an oval or circular shape, encircled by elongated ridges that gradually diminish toward the caudal field, and is typically positioned in the posterior region of the scale. This observation aligns with the findings of Devi, et al.^7^ for *N. japonicus*. Although the position of the scale focus can vary among fish species^18^, it is generally considered stable once formed and does not shift throughout the lifespan of the individual^73^. Teimori, et al.^74^ further indicated that the shape and size of the focus are influenced by a combination of genetic factors, environmental conditions, and developmental processes. Variation in focus morphology has been widely reported among different fish taxa, including triangular, rectangular, circular, and oval forms^1,3,56,75^.

The caudal region of *Nemipterus* scales lacks conventional microstructural features such as circuli and grooves; instead, it is distinguished by ornamentations composed of granulations (tubercles) and ctenii. Similar structural patterns have been documented in previous studies^3,7,15,45,51^. Additionally, the caudal field of the scales in the studied nemipterid species contained multiple pores, which likely function as minute canaliculi facilitating mucus secretion. This mucus layer may play an essential role in wound healing and in reducing hydrodynamic drag at the fish water interface, thereby enhancing locomotory efficiency^76^. Also, Teimori, et al.^77^ suggested that modification in the ornamentation of the posterior region may have hydrodynamic significance, and such features are subject to modification throughout ontogenetic development.

Previous studies have demonstrated the taxonomic significance of lateral line scales in fish classification^51,78–80^. Khalil, et al.^81^ reported substantial genus-level variability in the anterior–posterior placement of the lateral line tube in *Brycinus nurse* and *Alestes baremose*. In contrast, the *Nemipterus* species examined in the present study showed no distinct species-specific pattern in lateral line scale morphology.

In this investigation, the lateral line canal ran parallel to the anteroposterior axis of the scale, with posterior and anterior openings positioned medially within the focus region and separated from their respective scale margins. These observations correspond with the findings of Voronina and Hughes^78^, who examined more than 1,000 teleost species, including *N. zysron*, and classified such scales as Unmodified Tubular-Scalar I. In this type, the canal tube forms as an elevated structure on a fully developed elasmoid scale, with the tube and scale following independent developmental pathways^82,83^. The position of canal openings is considered an important diagnostic feature for differentiating taxa^6,11,79^.

In general, Fish scale morphology has been shown to be most effective for distinguishing species within the same family that exhibit differing body morphologies, moderately effective for differentiation within fish families, and least effective for separating distantly related taxa^17^. Variations in scale shape and structure are influenced by multiple environmental factors, including temperature, water currents, population density, food availability, and other ecological conditions^59,84^. In addition to environmental influences, genetic factors have also been highlighted as critical determinants of the taxonomic utility of scale characteristics^85^.

## Conclusions

This study demonstrates that scale morphology, together with traditional morphometric and geometric shape analyses, provides powerful tools for interspecific discrimination among *Nemipterus* species. The observed differences in scale characteristics, including surface ornamentation and structural features, underscore the value of scale-based assessments for species differentiation and stock identification. Collectively, these findings highlight the promising utility of scale morphology as a cost-effective approach for refining taxonomic classification and supporting conservation efforts. Nevertheless, classification accuracy may be enhanced by incorporating additional scale measurements and traits, such as scale thickness, flexibility, circuli counts, and lateral line canal patterns. Further comparative studies are required to validate the potential use of scale morphology and architectural design in species identification and phylogenetic applications within *Nemipterus* and related taxa.

## Data Availability

The data supporting this article are available from the corresponding author upon reasonable request.

## Abbreviations

BDFS: Beneath the anterior portion of the dorsal fin scales
POS: Post-operculum scales
BLLS: Beneath the lateral line, between the pectoral and pelvic fins scales
CPS: Caudal peduncle directly above the lateral line scales
ALLS: Anterior lateral line scales
MLLS: Middle lateral line scales
PLLS: Posterior lateral line scales
R1: Primary radii
R2: Secondary radii
L: Scale length
L1: Rostral field length
L2: Caudal field length
W: Scale width
